# Speed vs completeness: a comparative study of solitary and colonial tunicate embryogenesis

**DOI:** 10.3389/fcell.2025.1540212

**Published:** 2025-03-11

**Authors:** Chiara Anselmi, Katherine J. Ishizuka, Karla J. Palmeri, Paolo Burighel, Ayelet Voskoboynik, Kohji Hotta, Lucia Manni

**Affiliations:** ^1^ Department of Biology, Padova University, Padua, Italy; ^2^ Department of Biology, Stanford University, Palo Alto, CA, United States; ^3^ Institute for Stem Cell Biology and Regenerative Medicine, Stanford University, Stanford, CA, United States; ^4^ Department of Biosciences and Informatics, Keio University, Minato, Japan

**Keywords:** adultation, *Ciona robusta*, *Botryllus schlosseri*, colonial, evo-devo, heterochrony, solitary, tunicate

## Abstract

Solitary ascidians, such as *Ciona robusta*, have been used for over a century as model systems for embryological studies. These species are oviparous, producing many relatively small and transparent eggs, which are released and fertilized outside the parent body. Embryos develop rapidly in a stereotyped manner and reach the larva stage in less than 1 day (at 20°C). The larvae then settle and metamorphose into sessile juveniles in approximately 2 days. On the other hand, colonial ascidians are ovoviviparous, with heavily yolked eggs that develop inside the parent body. In the colonial *Botryllus schlosseri*, embryos are connected to the parental body via a cup-like placenta and develop into larvae within a week (at 20°C). These larvae, which possess both typical larval organs and prospective juvenile organs, are released into seawater, where they settle very rapidly, sometimes after only 15 minutes of free swimming. Then, they metamorphose into juvenile oozooids. The ability to study embryo development in colonial ascidians within the parent body is limited. To address this, we developed a method for *in vitro* culturing *B. schlosseri* embryos outside the parental body and combined it with time-lapse and confocal microscopy to describe the embryonic developmental stages. Moreover, we used histological analysis based on serial sections to investigate late-stage development, when embryo opacity made other techniques ineffective. We identified 19 stages of development, from the fertilized egg to the swimming larva, and described the stage of organ appearance and differentiation. Comparing the embryonic development timeline of *B. schlosseri* with that of *C. robusta*, we found heterochrony in development, particularly in the timing of organ appearance and growth rate. We hypothesize that this difference in maturation timing between solitary and colonial ascidians reflects a shift in the regulation of key developmental pathways that contributed to ascidian diversification. This heterochronic evolution likely facilitated a significant (approximately four-fold) shortening of the metamorphosis time in *B. schlosseri* by allowing embryos to remain in a safe ovoviviparous environment five times longer than those in *C. robusta* before hatching.

## 1 Introduction

Within chordates, tunicates exhibit the widest range of reproductive strategies. These marine invertebrates are considered the sister group of vertebrates (Delsuc et al., 2018), sharing several chordate features with them, such as the notochord, segmented musculature, pharyngeal pockets, endostyle/thyroid gland, and dorsal hollow neural tube. In colonial tunicates, some of these structures, like the endostyle and pharyngeal pockets, develop not only by passing through the typical pharyngula phylotypic stage (He et al., 2020) but also via stem cell-mediated processes during asexual reproduction or in whole-body regeneration (Laird et al., 2005; Voskoboynik et al., 2008; Alié et al., 2018; Manni et al., 2019). Other shared features between tunicates and vertebrates include secondary hair cell-like mechanoreceptors, neural crest-like cells, and embryonic proto-placodal areas (Patthey et al., 2014; Manni and Pennati, 2015; Anselmi et al., 2024; Todorov et al., 2024).

**FIGURE 1:**
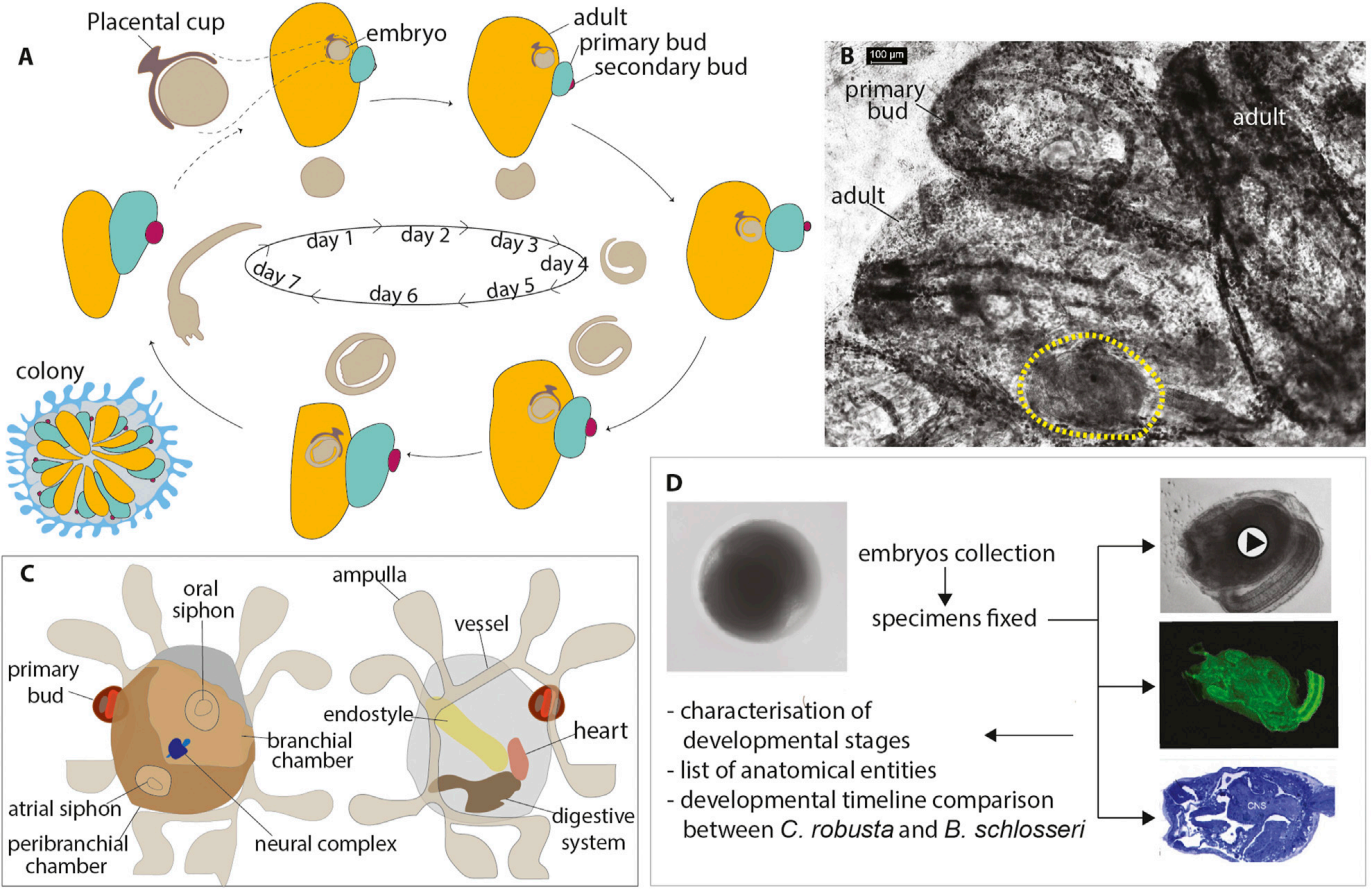
**(A)** B. *schlosseri* life cycle. A colony shows weekly (at 20°C) synchronized waves of budding accompanied by the regression and reabsorption of filtering adults (Manni et al., 2014). Blastozooids are organized in star-shaped systems embedded in a common tunic; a colonial circulatory system of vessels joins all the blastozooids, present in three generations: the adults, the primary buds growing on adults, and the secondary buds developing on the primary buds. Sexual and asexual cycles coincide: the embryo stage of development and the day of larval hatching in a colony are closely related to the colonial blastogenetic phase, so larvae are released before their parent resorption (Manni et al., 2007). **(B)** Details of an adult zooid (in ventral view) with an embryo (yellow dashed line) developing within the peribranchial chamber. Whole-mount colony. **(C)** Illustration of an oozooid in dorsal (left) and ventral (right) views showing main organs **(D)**. Methodological procedure followed to describe the development of *B. schlosseri*. Fertilized eggs and embryos across different developmental stages were gently removed from the parental peribranchial chamber. Samples were observed using a stereomicroscope, photographed, and fixed for CLSM and histology. Data analyses included the characterization of developmental stages, the list of anatomical entities and their definition, and the developmental timeline comparison between *C. robusta* and *B. schlosseri*.

Within ascidians, the main tunicate taxon, solitary species, such as *Ciona robusta*, are oviparous, producing large numbers of small, transparent eggs (140 μm in diameter), which are ovulated and fertilized by heterologous sperm in seawater, giving rise to embryos that develop autonomously from the parents (Figure 1A). These embryos rapidly (less than 1 day) reach the free-swimming tadpole larval stage (Hotta et al., 2007; Hotta et al., 2020). After swimming for several hours, the larvae adhere to a suitable substrate and undergo extensive metamorphosis, a process lasting a couple of days. During metamorphosis, prospective juvenile organs become recognizable, the body axis rotates 90°, and they complete their development, while larval tissues, including the tail and the brain, are resorbed. The resulting juvenile is a sessile, filter-feeding animal, which will continue to grow and increase in size throughout its entire life. In some species (the Enterogona ones), such as *C. robusta*, juveniles complete their development with the fusion of the two atrial siphon rudiments in a single dorsal structure during the post-metamorphosis period, 7 days after fertilization (Hotta et al., 2020). Generally, solitary ascidians have regenerative abilities restricted to the apical structures, such as the siphons and the brain (Vanni et al., 2022b). However, the Red Sea ascidian *Polycarpa mytiligera* is an exception; when a single individual is cut into three parts, each can regenerate into a whole body (Gordon et al., 2021). Due to *in vitro* fertilization and embryo culture, solitary ascidians have become model organisms to investigate cell lineage, blastomere determination, and morphogenesis. The ontology of anatomy and development is now available for *C. robusta*, standardizing developmental studies (Hotta et al., 2020). Ascidian embryogenesis is characterized by stereotyped development based on invariant early cell lineages and a remarkably small cell number (Kumano and Nishida, 2007). These unique features allow the study of chordate developmental programs at the cellular or even subcellular level using a variety of molecular tools, including CRISPR/Cas9 (Kogure et al., 2022; Pennati et al., 2024).

In contrast, colonial ascidians also reproduce asexually by budding (blastogenesis), producing zooids (blastozooids) through the involvement of pluripotent/multipotent stem cells (Alié et al., 2018; Manni et al., 2019, p. 20; Vanni et al., 2022a). These blastozooids form colonies of clonal individuals, all derived from cycles of blastogenesis originating from an individual (oozooid), which emerges from the metamorphosis of a larva and is considered the founder of the colony (Figure 1). Colonial ascidians exhibit extensive regenerative abilities (Vanni et al., 2022b). For example, in *Botryllus schlosseri* (Manni et al., 2014; Brunetti et al., 2017), which can be easily cultured on glass slides, whole-body regeneration is triggered when all the zooids are surgically removed from a colony due to pluripotent and/or multipotent stem and progenitor cells that initiate budding (Sabbadin et al., 1975; Voskoboynik et al., 2007; Tiozzo et al., 2008; Manni et al., 2019; Scelzo et al., 2019; Ricci et al., 2022; Vanni et al., 2022c).

Sexual reproduction in colonial ascidians occurs concomitantly with the asexual one. Typically, colonial species produce a few yolky eggs (in *B. schlosser*i, 1–3 eggs per zooid; Gasparini et al., 2015), which are larger and more opaque than those produced by solitary ascidians (Manni et al., 1993; Manni et al., 1994; Zaniolo et al., 1994b). Moreover, in colonial ascidians, both fertilization and embryogenesis occur within the parental zooids, with minimal or no parental nutrient contribution to the embryonic development (Zaniolo et al., 1987), except in rare viviparous species (*Botrylloides violaceus*, *Botrylloides lenis*, and *Hypsistozoa fasmeriana*) that develop from yolkless eggs and, therefore, rely on parental sustenance (reviewed in Zaniolo et al., 1998). In colonial ascidians, gestation lasts several days, and the resulting larvae are usually bigger than those produced by solitary ascidians. Moreover, they also display a variable level of adultation, *i.e.*, the early development of rudimentary adult organs, making them more complex than the larvae of solitary species. In *B. schlosseri*, for example, hatched larvae show open siphons, perforated protostigmata, the heart, the rudiment of the adult nervous system, and two buds (Kowarsky et al., 2021; Manni et al., 2022). Typically, in solitary ascidians, these structures (except buds) develop in the juvenile after the larva adhesion to the substrate. Therefore, there is heterochrony, *i.e.*, a change in the timing of developmental events (Keyte and Smith, 2014; Iwata and Vanderhaeghen, 2024), when comparing the embryonic and post-embryonic (*i.e.*, the post-metamorphic) development of colonial vs*.* solitary ascidians. This reflects a shift in the onset of growth and possibly the growth rate of some organs.

In *B. schlosseri*, attempts to fertilize isolated eggs (removed from the parent zooid) with isolated sperm and track their development *in vitro* were made in the past (Milkman and Borgmann, 1963; Milkman, 1967). However, their internal development and larval complexity hindered embryonic study, so a comprehensive description of whole-embryo development and cell lineage in a colonial ascidian is not yet available. Therefore, despite their heterochronic development, ascidians represent a valuable model for understanding the links between development and evolution, although comparative studies between solitary and colonial species development have been limited to detailed observations regarding specific organs (Manni et al., 2022). Nonetheless, due to the contemporary presence of different developmental strategies, *i.e.*, embryogenesis, blastogenesis, and whole-body regeneration, all resulting in similar individuals, colonial ascidians such as *B. schlosseri* provide an opportunity to investigate, from an evo-devo perspective, how different developmental trajectories generate the same adult form (Tiozzo et al., 2005; Manni and Burighel, 2006; Gasparini et al., 2011; Kowarsky et al., 2021).

This work aims to bridge the knowledge gap by presenting the first comprehensive description of the development and anatomy of a colonial ascidian embryo in *B. schlosseri*. These new data update previous observations on *B. schlosseri* embryogenesis, dating back a century (Scott, 1934; Grave, 1934; Grave and Woodbridge, 1924), and integrate information on the development of specific territories, such as the larval and adult nervous system, papillae, protostigmata, endostyle, heart, and hemocytes (Manni et al., 1999; Manni et al., 2004; Manni et al., 2022; Sorrentino et al., 2000; Tiozzo et al., 2005; Degasperi et al., 2009; Caicci et al., 2010; Gasparini et al., 2011; Gasparini et al., 2013; Kowarsky et al., 2021; Cima, 2023). Additionally, a comparative analysis of the developmental timing between *C. robusta* and *B. schlosseri* embryos reveals that heterochrony impacts not only alterations in the timing of development of some organs but also their rate of development.

## 2 Materials and methods

For *in vivo* observations and confocal imaging, mature *B. schlosseri* colonies were collected from piers at the Monterey Marina (CA, United States), close to the Hopkins Marine Station of Stanford University. The colonies were then attached to a glass slide and placed into an aquarium at 20°C (Boyd et al., 1986). Mature colonies for histological analysis were collected from floating blades of the marine plant *Zostera marina* in the Lagoon of Venice, near the Hydrobiological Station of the University of Padova (Chioggia, Italy). The colonies were removed from their natural substratum, made to adhere to glass slides, and maintained at 20°C (Sabbadin, 1955).

### 2.1 Preparation of embryos for time-lapse imaging

Embryos and developing larvae, which are visible inside the parental body and are individually suspended in a placental cup within the peribranchial chamber (Zaniolo et al., 1987), were removed using a thin needle. Embryos in a colony develop at the same rate. Therefore, multiple synchronized embryos were obtained from a single colony; however, several mature colonies at different blastogenetic phases were necessary to obtain embryos at different stages. After carefully removing the embryos from the parental organism with an insulin needle, they were transferred using a P200 pipette into Petri dishes containing filtered seawater. To optimize the development of each embryo, a maximum of 25 embryos were placed at the center of each dish. This approach ensured that the embryos had sufficient space for growth while minimizing interference between them. The seawater was filtered using a Millipore syringe filter (pore size 0.22 μm) to maintain a clean and contaminant-free environment, which was essential for the embryos’ healthy development. The water in the Petri dishes was changed daily using a glass pipette, which allowed for the precise removal of waste products and the addition of fresh, filtered seawater, ensuring a stable and optimal culture medium. The embryos were reared at a constant temperature of 23°C, which was determined by the environmental conditions of the BZ-9000 Keyence Microscope used for observation. Due to technical limitations, the temperature could not be independently adjusted from the microscope setting. Throughout the developmental process, the embryos were closely monitored under the microscope, which provided high-resolution imaging for detailed observation of their growth and developmental stages.

### 2.2 Image acquisition at confocal scanning laser microscopy

Embryos were fixed for 30 min at room temperature with 4% paraformaldehyde in MOPS buffer (0.1 M 3-(N-morpholino) propane sulfonic acid), adjusted to a pH value of 7.5, and washed in PBT two times. Fixed samples were stained for 30 min in 1/1,000 diluted cell mask orange for staining cytoplasm. After three washes with PBT, Alexa phalloidin 546 was used for actin staining overnight at 4°C. Samples were made transparent by dehydrating them with a series of solutions of 2-propanol in PBT, followed by treatment with BABB (benzyl alcohol (Sigma B-1042)/benzyl benzoate (Sigma B-6630) in a 1:2 ratio). For nuclear staining, embryos were fixed, stained with DAPI (Vector Laboratories), and mounted in a mounting medium (VECTASHIELD). Stained samples were observed using a confocal laser microscope (Olympus FV1000) under a ×10–×40 oil objective lens. Three-dimensional images were reconstructed from stack images (interval 1–3 μm) using Imaris software. Several dozen embryos were collected for each stage, and representative embryos were selected for imaging.

### 2.3 Histology

Five embryos, both in the tailbud and larva periods, were fixed for 2 h in 1.5% glutaraldehyde in 0.2 M sodium cacodylate and 1.6% NaCl buffer. After three washes in 0.2 M sodium cacodylate and 1.6% NaCl buffer, samples were post-fixed for 30 min in 1% OsO_4_ in 0.2 M cacodylate buffer at 4°C. The samples were dehydrated and subsequently soaked in Epon and propylene solution. They were then embedded in resin at 37°C, 45°C, and 60°C, oriented, and sectioned using a Leica Ultramicrotome. Sections, 1-μm-thick, were stained with toluidine blue.

### 2.4 Whole-mount preparations

Colonies adhering to glass slides were anesthetized with MS 222, fixed in Bouin’s fluid, washed in PBS, and stained with Mayer’s hemalum (Sigma-Aldrich, MHS32). After washing in distilled water, the colonies were dehydrated in ethanol, cleared in xylene, and mounted with Technovit 8100 (EMS cat. no. 14,654).

## 3 Results

### 3.1 Embryo development in *B. schlosseri*

#### 3.1.1 *B. schlosseri* embryos can survive and develop outside the parental body

**FIGURE 2:**
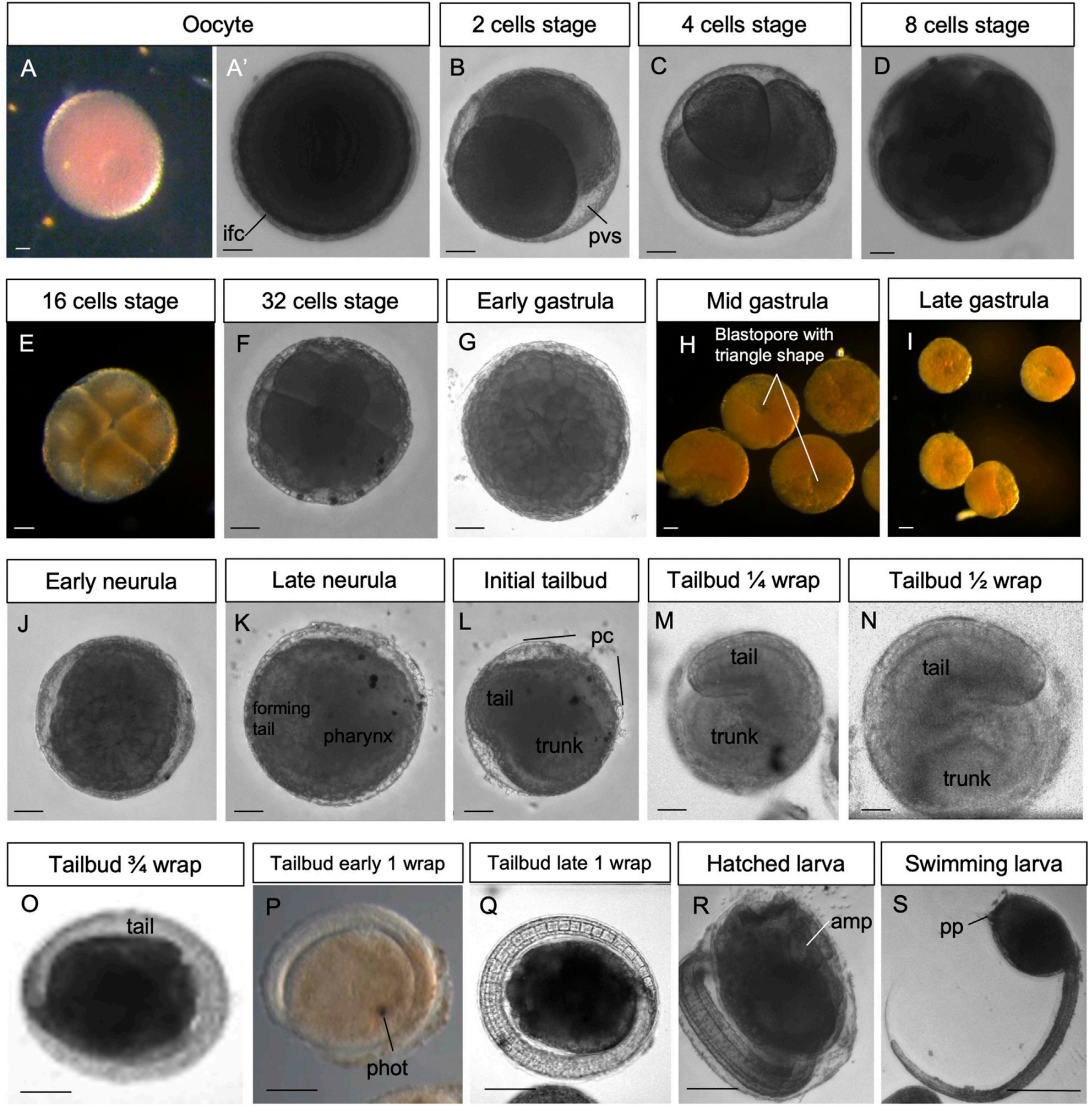
**(A–S)** *In vivo* embryos at stages 1–19. Stereomicroscopy. Scale bar, 100 μm. amp, ampullae; ifc, inner follicles cells; pc, placental cup; phot, photolite; pp, papilla; pvs, perivitelline space. See list of abbreviations in Supplementary Table S1.

**FIGURE 3:**
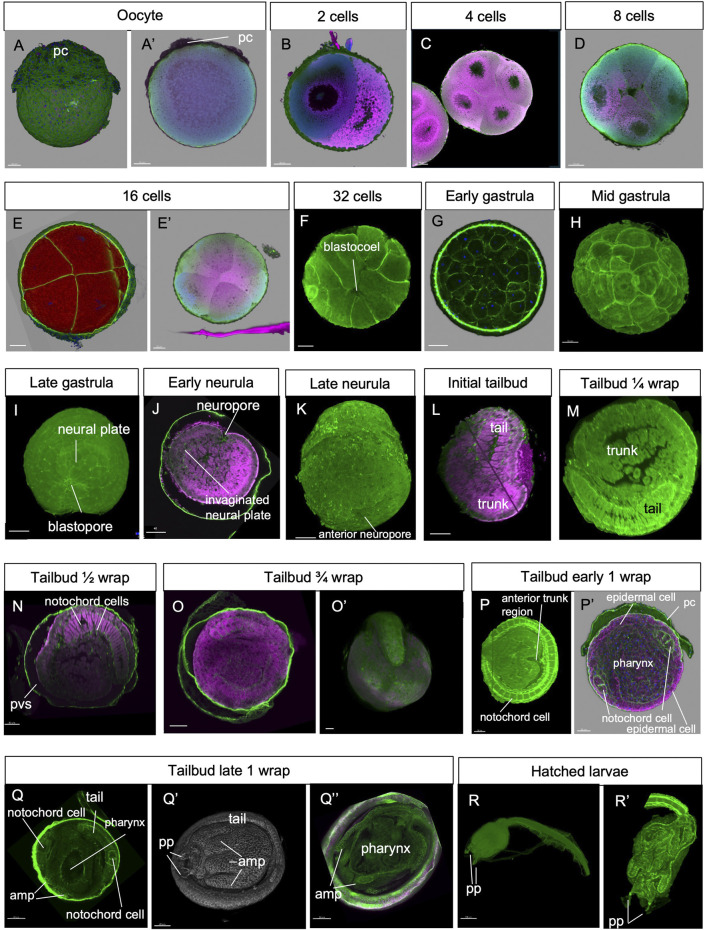
**(A–R)** Confocal laser scanning microscopy of embryos at stages 1–19 (green: Alexa phalloidin; pink: cell mask orange). amp, ampullae; pc, placental cup; pp, papilla; pvs, perivitelline space.

To study embryogenesis in *B. schlosseri*, we analyzed the development of embryos *in vitro* by dissecting them from the parental colony (Figure 1B; Table 1). We successfully cultured these embryos and tracked their development until the oozooid stage (Kowarsky et al., 2021). Unlike solitary ascidians, where stages can be easily defined based on *in vivo* imaging due to embryo transparency (Hotta et al., 2007), *B. schlosseri* required additional imaging techniques to observe its development. Using a combination of *in vivo* imaging (observations and movies) and confocal scanning laser microscopy (CLSM) (virtual sections and 3D reconstructions), we were able to define the stages of embryogenesis, from the zygote to the swimming larva. These stages correspond to the meta-periods “Pre-embryonic development” and “Embryonic development, pre-metamorphosis” (Table 2; Figures 2, 3; Supplementary Video S1) (Hotta et al., 2007). This combination of methods allowed us to identify a higher number of stages compared to our previous study (Kowarsky et al., 2021).

**TABLE 1:** Number of reared embryos per stage and percentage of embryos completing development. The “total number of oozooids” indicates the number of embryos that passed through the metamorphosis stage becoming filter-feeding oozooids, even if not normal.

Colony ID	Developmental period/stage of embryo removal from colony	Number of removed embryos	Total number of oozooids	Number of normal oozooids	% of embryos completing development	% of embryos with normal development
2.0	2 cells	16	5	3	31.25%	18.75%
2.1	2 cells	18	4	2	22.2%	11.1%
7	16 cells	25	20	2	80%	8%
3	After 16 cells until early gastrula	3	2	0	66.67%	0
6	Early gastrula	10	4	2	40%	20%
9	Early neurula	18	8	2	44.4%	11.1%
1	Early neurula	11	7	2	63.6%	18.1%
4	Tailbud late 1 wrap	25	10	3	40%	12%
5	Tailbud late 1 wrap	25	20	17	80%	68%
12	Tailbud late 1 wrap	20	10	3	50%	15%

**TABLE 2:** Timetable of the development of *B. schlosseri* embryo referred to the meta-period “Embryonic development, pre-metamorphosis”. “Stage (20°C)” refers to the stage nomenclature proposed in Kowarsky et al. (2021): E indicates the embryonic development; the number following it (1–7) indicates the day of the blastogenetic cycle in which the stage was found; the last number (separated by dot from the previous one), when present, numbers the embryo stage in that day. The stages E6–E7.1 and E6–E7.2 last approximately 2 h and depend on colony takeover onset; they occur after 6–7 days from the oral siphon aperture in adults, event allowing the fertilization of ovulated eggs. *, new stages with respect to Kowarsky et al. (2021). “Stage N” indicates the progressive stage number according to the practice used for solitary ascidians (Hotta et al., 2007; Hotta et al., 2020). Hpf, hours post fertilization. Note that the *in vitro* development was faster than *in vivo* since it occurred at a higher temperature. The percentage of development is calculated referring to the *in vitro* development at 23°C, with 0% of development occurring at day 1 (zygote) and 100% at day 4.5 (108 hpf) (hatched larva).

Stage (20°C)	Stage N°	Stage name	Definition	Hpf *in vitro* development (23°C)	% of development
I. Zygote period					
E1.0	1	1 cell	Zygote, fertilized egg	0–1 h	0
II. Cleavage period					
E1.1	2	2 cells	Two-cell stage embryo	1 hpf	0.9
E1.2	3	4 cells	Four-cell stage embryo	2 hpf	1.8
E1.3	4	8 cells	Eight-cell stage embryo	4 hpf	3.7
E1.4	5	16 cells	16 cells of different sizes	6 hpf	5.5
E1.5	6	32 cells	32-cell stage embryo, blastula as hollow sphere of cells	8 hpf	7.4
III. Gastrula period					
E2.1*	7	Early gastrula	Sinking of the embryo vegetal side; large blastopore	16 hpf	14.8
E2.2*	8	Mid gastrula	Invagination of mesodermal tissue occurring and blastopore with triangle shape	18 hpf	16.6
E2.3*	9	Late gastrula	Closing blastopore	23 hpf	21.3
IV. Neurula period					
E3.1*	10	Early neurula	Neuropore (blastopore) halfway in embryo; notochord cells recognizable but not yet in convergent extension	25 hpf	23.1
E3.2*	11	Late neurula	Pear-shaped embryo with anterior neuropore. Embryo elongating	29 hpf	26.8
V. Tailbud period					
E3.3*	12	Initial tailbud	Initial separation between tail and trunk. Neural tube cylindrical	31 hpf	28.7
E3.4*	13	Tailbud ¼ wrap	Tail circumscribing ¼ of the trunk; embryo resembling a comma	35 hpf	32.4
E3.5*	14	Tailbud ½ wrap	Tail circumscribing half of the trunk. Notochord cells at the end of convergent extension movement	39 hpf	36.1
E4	15	Tailbud ¾ wrap	Tail circumscribing ¾ of the trunk	40 hpf	37
E5	16	Tailbud early 1 wrap	Tail making one complete turn around the trunk	44 hpf	40.7
E6	17	Tailbud late 1 wrap	Tail encircling the trunk more than 1 wrap (maximum extension: 1.5 wrap). Trunk ovoidal and increased in size; papillae recognizable	66 hpf	61.1
VI. Larva period					
E6–E7.1*	St. 18	Hatched larva	Larva swimming upward, attracted toward light sources	4–5 days	100
E6–E7.2*	St. 19	Swimming larva	Larva first indifferent to light, then negative to light, and touching the substrate repeatedly, before attaching permanently to the substrate and beginning to metamorphose	4–5 days	

In general, embryos removed at early developmental stages (up to the neurula period) had a lower survival rate through metamorphosis than those removed at later stages. For example, only 20% of embryos removed during the gastrula period (stages E2.1–E2.3 in Table 2) reached the oozooid stage and opened their siphons. In contrast, 68% of embryos removed during the tailbud period (Stage E6 in Table 2) completed development normally. However, in several cases, oozooids died soon after metamorphosis, largely due to abnormal ampulla (Figure 1C) development and the inability to adequately attach to the substrate. Therefore, the final number of healthy oozooids was significantly lower than the total number of oozooids obtained from metamorphosed larvae.

#### 3.1.2 Timetable and description of *B. schlosseri* embryonic development

To describe the meta-periods “Pre-embryonic development” and “Embryonic development, pre-metamorphosis,” we estimated the timing of development based on the colonial blastogenetic phases (Scott, 1934; Manni et al., 2007) as we were unable to determine the exact time of fertilization, which occurs inside the parental body shortly after siphon opening (Milkman, 1967). Since the adult life span is 6–7 days at 20°C (the same as embryonic development until larval hatching; Manni et al., 2007), we referred to the development stages using the formula “E.1,” “E.2,” and so on, where E stands for embryo and the number following it indicates the day of development, following the approach of Kowarsky et al. (2021). Without precise reference to the time of fertilization, the hatching could occur after 6 or 7 days of larval development. It is also to be considered that the larval swimming phase in *B. schlosseri* is very short, and settling occurs, on average, after a couple of hours of free swimming (sometimes, even after only 15 minutes of free swimming, according to Grave and Woodbridge, 1924). For these reasons, the start and duration of the larva period were defined as “E.6–7.1” and “E.6–7.2” in this study.

The *in vitro* development was faster than the *in vivo* one since it occurred at a higher temperature (23°C, Table 2); this is consistent with previous observations comparing the duration of the blastogenetic cycle at different temperatures (Gasparini et al., 2015).

The subdivision into periods and stages is in line with the developmental ontology published for *C. robusta* (Hotta et al., 2007). Only early development up to the 32-cell stage (cleavage period) was easily identifiable *in vivo*. After this stage, the presence of yolk within blastomeres rendered embryos opaque, making it difficult to track each mitosis. Gastrulation was identified by the appearance of the blastopore, while neurulation was marked by the presence of the neural plate and the forming neural tube. During the tailbud period, the main parameter used to define the stages was the length of the tail, which grows on the left around the trunk at its equatorial level beneath the chorion and, at maximum extension, wraps around the trunk 1.5 times (Manni et al., 1999; Kowarsky et al., 2021). These stages deeply differ from those described in *C. robusta* (Hotta et al., 2007).

The detailed description of tailbud and larva periods is primarily based on histological analysis of serial sections of whole embryos sectioned according to different planes as the opacity of the late embryo also prevented deep laser penetration at CLSM. The list of the anatomical entities peculiar to *B. schlosseri* recognized in this study, along with their definitions, is provided in Supplementary Table S1; it represents a revision of the anatomical entity list published for the juvenile of *C. robusta* (Hotta et al., 2020).

#### 3.1.3 Meta-period: pre-embryonic development

##### 3.1.3.1 Period: pre-fertilization

*Stage: Unfertilized egg* (Figure 2A’, Figure 3A-A′). The ovulated egg contains densely packed yolk globules and measures approximately 250–300 μm in diameter. A continuous thin layer of inner follicular cells, which collaborate with the oviduct cells to form the placental cup, covers it (Zaniolo et al., 1987). The inner follicle cells lie on the acellular vitelline coat (or chorion). Within the perivitelline space, (individuated between the oolemma and the vitelline coat), several test cells are present. The outer follicle cells that surrounded the oocyte during oogenesis were discharged at ovulation, remaining in the mantle as a sort of *corpus luteum* (Zaniolo et al., 1987).

##### 3.1.3.2 Meta-period: embryonic development, pre-metamorphosis

###### 3.1.3.2.1 I period: zygote

*Stage E1.0* (Stage 1, day 1 of development). The zygote (1-cell embryo) consists of a single fertilized cell. The stage extends from fertilization to the completion of the first mitotic cycle.

###### 3.1.3.2.2 II period: cleavage

*Stage E1.1* (Stage 2, day 1 of development) (Figures 2B, 3B): 2-cell embryo. The first division separates the left and right halves of the embryo.

*Stage E1.2* (Stage 3, day 1 of development) (Figures 2C, 3C): 4-cell embryo. The second cleavage plane is determined by the embryos dividing into anterior and posterior halves.

*Stage E1.3* (Stage 4, day 1 of development) (Figures 2D, 3D): 8-cell embryo. The third cleavage plane is horizontal and separates the animal from the vegetal blastomeres. At this stage, the four founder lineages are defined as follows: A, anterior vegetal; B, posterior vegetal; a, anterior animal; b, posterior animal (Conklin, 1905).

*Stage E1.4* (Stage 5, day 1 of development) (Figures 2E, 3E’): 16-cell embryo. The embryo possesses groups of cells of different sizes that are clearly recognizable. The animal and vegetal cells have undergone the fourth cleavage, and blastomeres show bilateral symmetry in their arrangements. During these early stages, embryos are characterized by a spherical shape and pink color (brown under a stereomicroscope).

*Stage E1.5* (Stage 6, day 1 of development) (Figures 2F, 3F): 32-cell embryo, blastula. The embryo is a hollow sphere of cells. From this stage until gastrulation, blastomeres continue dividing, but the number of cell divisions is no longer detectable through *in vivo* observations under a stereomicroscope.

### 3.2 Period: gastrula

*Stage E2.1* (Stage 7, day 2 of development) (Figures 2G, 3G): early gastrula stage. The invagination of the endodermal layer begins, and a large blastopore is recognizable.

*Stage E2.2* (Stage 8, day 2 of development) (Figures 2H, 3H): mid gastrula stage. Gastrulation continues with the involution of mesodermal cells. The blastopore has a triangle shape.

*Stage E2.3* (Stage 9, day 2 of development) (Figures 2I, 3I): late gastrula stage. The blastopore is closing, and the neural plate is forming.

#### 3.2.1 Period: neurula

*Stage E3.1* (Stage 10, day 3 of development) (Figures 2J, 3J): early neurula stage. The larval neural plate forms the neural fold; the nervous system closes, and the anterior neuropore (blastopore) is open in the embryo. The notochord has not yet started the process of convergent extension.

*Stage E3.2* (Stage 11, day 3 of development) (Figures 2K, 3K): late neurula stage. The embryo is oval. The neural tube is closed. The pharynx has an oval lumen delimited by endodermal cells. In the forming tail, the endodermal strand is recognizable ventral to the notochord, which is oval. Small mesodermal cells, representing the muscle cell precursors, flank the notochord cells.

#### 3.2.2 Period: tailbud

**FIGURE 4:**
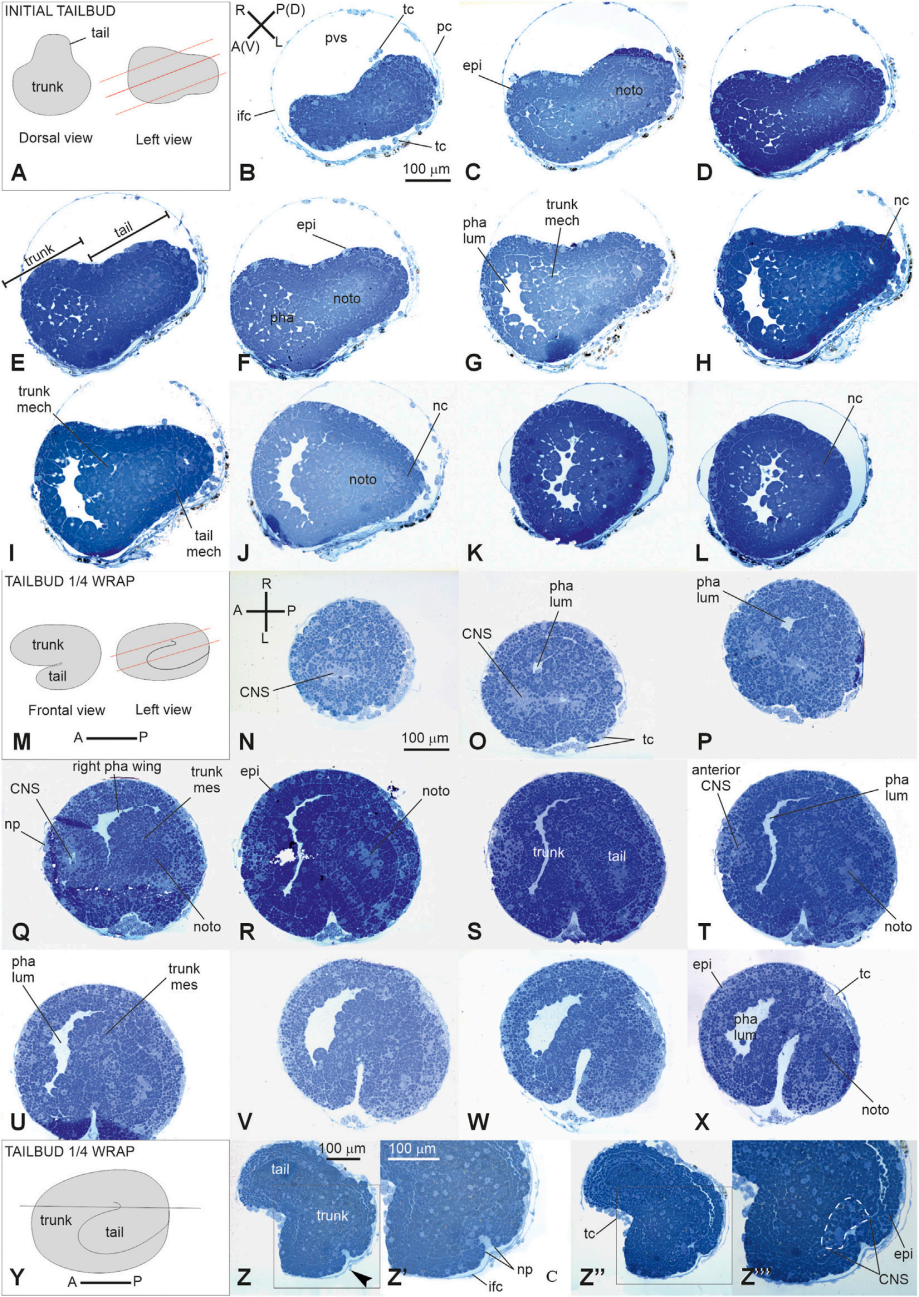
V period. **(A–L)** tailbud. Stage E3.3 (Stage 12, day 3 of development). Initial tailbud **(M–Z‴)**: *Stage E3.4* (Stage number 13, day 3 of development). Tailbud ¼ wrap **(A)**. Dorsal and lateral illustrations of embryos at the initial tailbud stage. Red lines indicate the cut planes in lateral view **(B–L)**. Frontal-transverse sections selected from a complete series of the same embryo cut from the posterior **(P)** side to the anterior **(A)** one, slightly tilted dorso **(D)**-ventrally **(V, M)**. Frontal and lateral illustrations of embryos at tailbud ¼ wrap. Red lines indicate the cut planes in lateral view **(N–X)**. Frontal serial sections selected from a complete series of the same embryo cut from the dorsal-right side **(N)** to the ventral-left one **(X, Y)**. Illustration of an embryo at tailbud ¼ wrap; the red line indicates the cut planes in left view **(Z-Z**’’’**)**. Two frontal sections **Z,** one more dorsal than the **(Z’’)** selected from a complete series of the same embryo at the neuropore level. **(Z’)** and **(Z’’’)** represent enlargements of the square areas shown in **(Z)** and in **(Z’)**, respectively. White dotted line in **(Z‴)** represent the central nervous system. **(A–P)** and **(R–L)** Illustrations of anterior–posterior and right-left axes, respectively; CNS, central nervous system; epi, epidermis; ifc, inner follicle cell; mech, mesenchyme cell; nc, nerve cord; noto, notochord; np, neuropore; pc, placental cup; pha, pharynx; pha lum, pharynx lumen; pvs, perivitelline space; righ pha wing, right wing of the pharynx; tc, test cell; trunk mes, trunk mesenchyme. Enlargement is the same in **(B–L)**, **(N–X)**, **(Z, Z’)**, and **(Z’, Z’’’)**. Toluidine blue.

*Stage E3.3* (Stage 12, day 3 of development) (Figures 2L, 3L, 4A–L): initial tailbud. The embryo is pear-shaped, and the first separation between the tail and the trunk appears. Different embryonic tissues are progressively recognizable at the histological level due to the different cell shapes, sizes, and arrangements, as well as their spatial relationships. Epithelia are monolayers. The entire embryo is covered by small epidermal cells (Figure 4F). In the trunk, the nervous system is in its typical dorsal position (Figure 4H); it has cells that are smaller than the endodermal ones. The anterior neuropore is present. In the tail, which is straight, the nerve cord elongates dorsal to the notochord (Figure 4J). The latter is ovoid, and 2–4 cells can be recognized in the same cross section. Endodermal cells are very rich in yolk and form the pharynx rudiment (branchial chamber rudiment) in the trunk, which is more recognizable in the anterior trunk than in the posterior trunk. The pharynx exhibits a small, oval lumen (Figure 4G). Mesenchyme cells occupy spaces between the epidermal and endodermal leaflets in the ventral and lateral trunks (Figure 4G). The yolk is distributed in all the embryo cells, decreasing in quantity from the notochord and endodermal cells to mesenchymal cells and nervous system cells.

*Stage E3.4* (Stage 13, day 3 of development) (Figures 2M, 3M, 4M–Z’’‘): tailbud ¼ wrap. A thick tail circumscribes ¼ of the trunk equatorially on its left side, and the embryo resembles a comma (Figure 4S). The tail, pressing against the trunk’s left side, slightly deforms the pharynx lumen, posteriorly wider on the right side than on the left side. The anterior neuropore is closed (Figure 4Z’’); its position is marked by a slight ectodermal depression.

**FIGURE 5:**
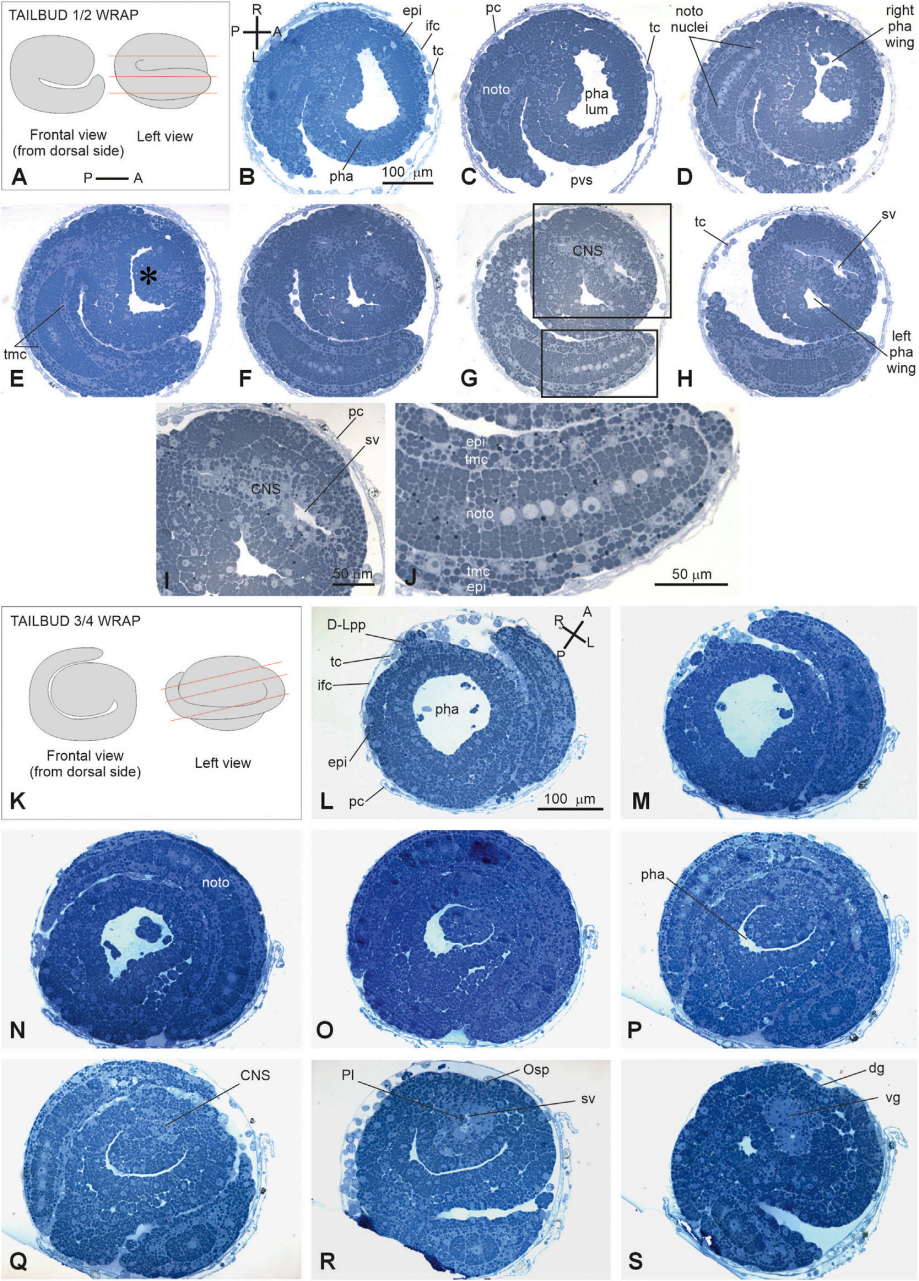
**(A–H)**: V period. Tailbud 1/2 wrap. Stage E3.5 Stage 14, day 3 of development A. Frontal and left illustrations of embryos. Red lines indicate the cut planes in lateral view **(B–J)**. Sagittal-oblique serial sections selected from a complete series of the same embryo cut from the dorsal-right side to the ventral-left side. **(E)** Wide depression on the pharynx roof (asterisk) caused by the sensory vesicle expansion. Squared areas in G are enlarged in I and J to show details of the sensory vesicle and of the tail **(K–S)**: V period. Tailbud ¾ wrap. Stage E4 (Stage 15, day 4 of development). Frontal and left illustrations of embryos. Red lines indicate the cut planes in lateral view **(K)**. Frontal and left illustrations of embryos. Red lines indicate the cut planes in lateral view **(L–O)**. Frontal-oblique sections selected from a complete series of the same embryo cut from the ventral-left-anterior side to the dorsal-right-posterior side. In **(B, L)**, **(A–P)** and **(R–L)** show the anterior–posterior and right–left axes, respectively; CNS, central nervous system; dg, dorsal groove; **(D–L)** pp, dorsal left papilla; epi, epidermis; ifc, inner follicle cells; left pha wing, left wing of the pharynx; nc, nerve cord; noto, notochord; Osp, oral siphon primordium; pc, placental cup; pha, pharynx; pha lum, pharynx lumen; Pl, photolith; pvs, perivitelline space; right pha wing, right wing of the pharynx; sv, sensory vesicle; tc, test cell; tmc, tail muscle cell precursor; vg, visceral ganglion. Enlargement is the same in **(B–H)**, **(I–J)**, and **(L, S)**. Toluidine blue.

*Stage E3.5* (Stage 14, day 3 of development) (Figures 2N, 3N, 5A–J): tailbud ½ wrap. The tail, now thinner than in the previous stage, circumscribes half of the trunk (Supplementary Figure S5). A total of 36 notochord cells are in convergent extensions, assuming a disc shape and arranging in a single line (Figure 5J). Three symmetrical lines of muscle cell precursors flank them. The nervous system begins to expand anteriorly in the sensory vesicle (identifiable due to its lumen) (Figure 5H) that presses on the pharynx roof (Figure 5E), narrowing the pharynx lumen on its right side. The pharynx is also deformed on its left side by the tail and posteriorly by mesenchymal cells in the ventral right trunk.

*Stage E4* (Stage 15, day 4 of development) (Figures 2O, 3O’, Figure 5K–O): tailbud ¾ wrap (tail circumscribing ¾ of the trunk (less than one complete wrap)). Three small anterior papillae, two dorsal and one ventral, protrude anteriorly (Figure 5L). The sensory vesicle with the photolith (the pigmented organ responding to both gravity and light; Sorrentino et al., 2000; Figure 5N), the visceral ganglion (Figure 5O), the neck, and the nerve cord are recognizable. Dorsally, the ectoderm deepens in the dorsal groove (Figure 5O), extending antero-posteriorly in the middle ectoderm: initially, it is in the form of a wide depression. The pharynx is larger than in the previous stage; it is depressed dorsally by the sensory vesicle and on the left by the growing tail. In the tail, the notochord cells are located in a single line, ventral to the nerve cord. Muscle cell precursors (still without evident myofibrils) are organized in three symmetric lines of cells flanking the notochord.

**FIGURE 6:**
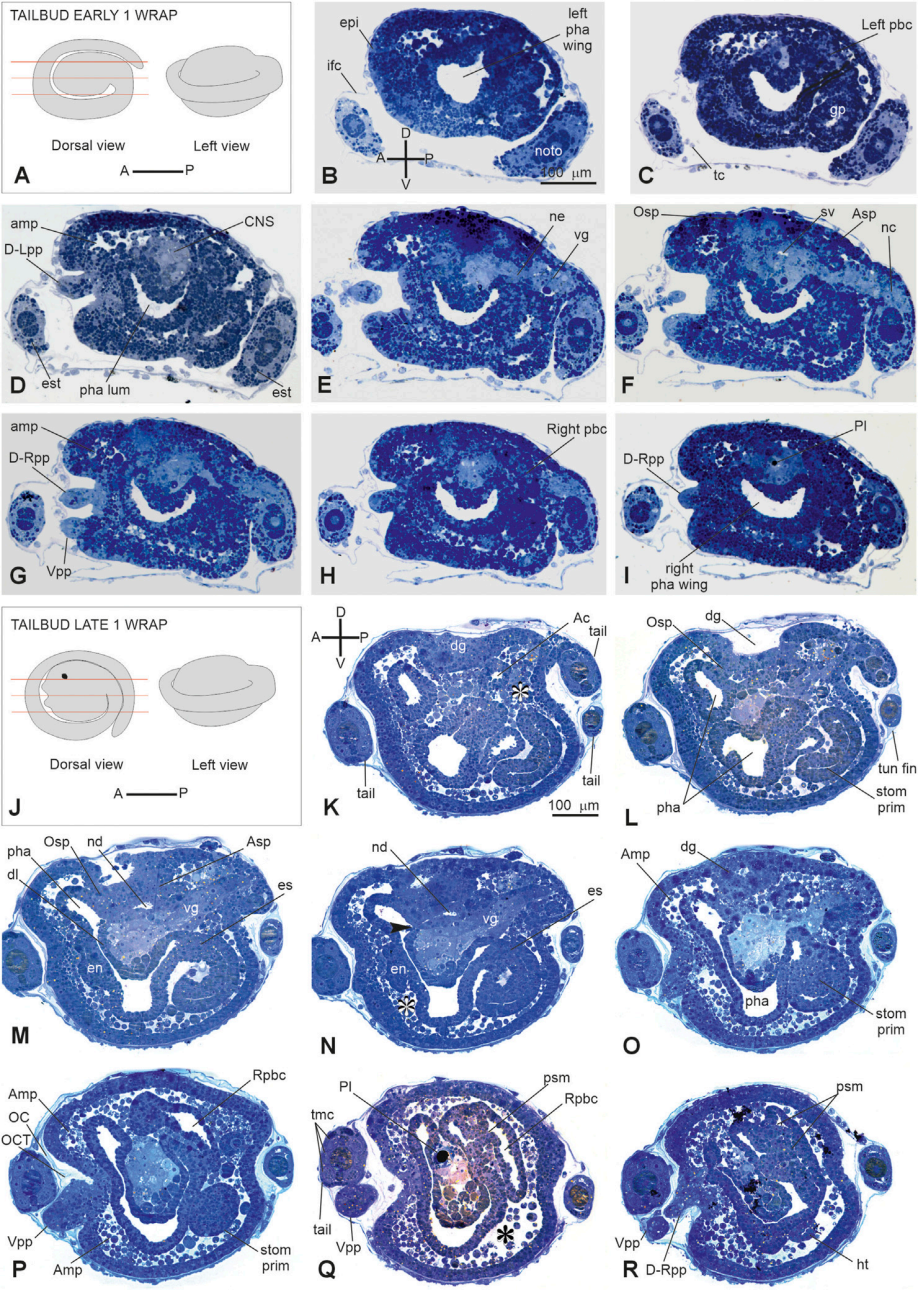
**(A–I)**: V period. Tailbud early one wrap. Stage E 5 Stage 16, day 5 of development. A. Frontal and left illustrations of embryos. Red lines indicate the cut planes in dorsal view **(B–I)**. Sagittal serial sections selected from a complete series of the same embryo cut from the left side to the right side. **(F)** Areas individuating the oral (Osp) and atrial (Asp) siphon primordia. Both the ampullae (amp in **D**, **G**) and the papillae (D-Rpp and Vpp in G) protrude from the anterior epidermis **(J–R)**: V period. Tailbud late one wrap. Stage E6 (Stage 17, day 6 of development). **(J)** Frontal and left illustrations of embryos. Red lines indicate the cut planes in the dorsal view **(K–R)**. Sagittal sections selected from a complete series of the same embryo cut from the left side to the right side. The dorsal groove (dg in **H–O**), the neurohypophyseal duct (nd in **M–N**), and the oral (Osp in **L**) and atrial (Asp in **M**) siphon primordia are well-recognizable. In B and K, **(A–P)** and **(D–V)** show the anterior–posterior and dorsal-ventral axes, respectively; asterisks in (**K**, **N**, and **Q**) represent blood lacuna; arrowhead in N represents neurohypophyseal duct aperture in the pharynx lumen. Ac, atrial cavity; Amp, ampulla; Asp, atrial siphon primordium; CNS, central nervous system; dg, dorsal groove; D-Lpp, dorsal left papilla; D-Rpp, dorsal right papilla; en, endostyle primordium; epi, epidermis; es, esophagus; est, endodermal strand; ht, heart; ifc, inner follicle cells; left pbc, left peribranchial chamber; left pha wing, left wing of the pharynx; nc, nerve cord; ne, neck; noto, notochord; OC, outer cuticular layer of tunic; OCT, outer compartment of tunic; Osp, oral siphon primordium; pha, pharynx; pha lum, pharynx lumen; Pl, photolith; psm, protostigma; Rpbc, right peribranchial chamber; righ pha wing, right wing of the pharynx; stom prim, stomach primordium; sv, sensory vesicle; tmc, tail muscle cell precursors; tun fin, tunic fin; vg, visceral ganglion; Vpp, ventral papilla. Enlargement is the same in **(B–I)**, and in **(K, R)**. Toluidine blue.

*Stage E5* (Stage 16, day 5 of development) (Figures 2P, 3P’, Figure 6A–I; Supplementary Figure 2A–C): tailbud early one wrap. The embryo trunk maintains a relatively circular shape, and the tail makes one complete turn around the trunk. The three papillae are well evaginated, without a cavity (Figures 6D, G); the interpapillary region is depressed. Close to the papillae, a ring of eight ampulla rudiments is present. Anteriorly, the pharynx rises in front of the sensory vesicle, toward the dorsal ectodermal invagination of the dorsal groove, representing the oral siphon primordium (stomodeum) (Supplementary Figure 2A); posteriorly, the pharynx extends into the esophagus and the stomach. The atrial chamber rudiment is in the form of a single dorsal ectodermal invagination in communication with the outside (Supplementary Figure 2B). In the brain, a small sensory vesicle, containing the forming photolith, is recognizable. The neurohypophyseal duct, representing the rudiment of the oozooid neural complex (Manni et al., 1999), is open into the pharynx. The heart is in the form of a compact mass of mesodermal cells. In the hemocoel, hemoblasts (lymphocyte-like cells) and morula cells can be detected (Supplementary Figure 2B–C; Kowarsky et al., 2021).

*Stage E6* (Stage 17, day 6 of development) (Figures 2Q, 3Q-Q″, Figure 6K–R): tailbud late one wrap. The tail encircles the trunk more than one wrap; it will continue growing to reach its maximum extension, making 1.5 wraps. The trunk is ovoid and progressively increases in size. Wide blood lacuna and sinuses are recognizable since epithelia are thinning for yolk consumption and the trunk is enlarging. The body wall (Supplementary Table S1) is rich in hemocytes. Phagocytic cells (hyaline amebocytes) and pigment cells can be detected. The larval tunic (outer cuticular layer and outer compartment), which constitutes the larval fins and will be lost at metamorphosis, is present around the trunk and the tail. The three papillae are more protruded anteriorly than in the previous stage, and their receptor end-organs are forming (Supplementary Figure 2D–E). The eight blood ampullae are well expanded. Dorsally, the dorsal groove occupies approximately 1/3 of the trunk length, and the tunic fills it; at this stage, the oral and the atrial siphons are developing (Figure 6M). The atrial chamber rudiment loses its communication with the outside at the end of this stage. Later, it elongates posteriorly in two wide invaginations, assuming a horseshoe shape; these invaginations descend, flanking the neural tube. Their bottom, on the right and left, represents the rudiments of the peribranchial chambers, which are derived from the uneven atrial chamber rudiment. A small left ganglionic vesicle is in continuity with the neurohypophyseal duct (Figures 6M, N; Manni et al., 1999). Its anterior-most part is in the form of a small duct opening into the pharynx. The neurohypophyseal region, posterior to the duct, is involved in the delamination of neuroblasts to form the adult cerebral ganglion (Manni et al., 1999; Sorrentino et al., 2000). The sensory vesicle is on the right of the visceral ganglion and contains an evident photolith (Figure 6Q; Manni et al., 1999). The branchial chamber is more deformed than in the previous stage. It exhibits a flat endostyle primordium on its floor, with two lateral–dorsal wings embracing the visceral ganglion. Two to three protostigmata are perforated, following an anterior–posterior pattern that allows the communication between the pharynx lateral–dorsal wings and the posterior descending peribranchial chambers. After the larva adhesion, the oozooid will exhibit five long protostigmata, dorsoventrally oriented. The peribranchial chambers are ventrally elongated; on the left, the peribranchial chamber is close to the stomach to form the perivisceral epithelium surrounding the gut. The latter grows dorsally and closes the dorsal groove in the region where the atrial siphon is opening. The heart is a hollow vesicle that begins to invaginate along its raphe (Figure 6R). As this stage progresses, an additional layer of the tunic is also recognizable around the trunk: the inner compartment of the tunic, with its cuticle (inner cuticular layer) representing the definitive tunic of the post-metamorphosing oozooid (Supplementary Figure 2D–F). The sensory vesicle sits on the right of the visceral ganglion. The neurohypophyseal duct parallels the dorsal groove and separates from the left ganglionic vesicle. The dorsal lamina (Figure 6M) is recognizable on the roof of the pharynx. The left peribranchial epithelium follows the gut growth, enveloping it as perivisceral epithelium; the gut is completely formed.

##### 3.2.2.1 Period: swimming larva

This period lasts 2 hours on average (Grave and Woodbridge, 1924) and is divided into two short stages. These stages are characterized by different larval behaviors and different conformations of the anterior papillae (Grave and Woodbridge, 1924; Caicci et al., 2010).

**FIGURE 7:**
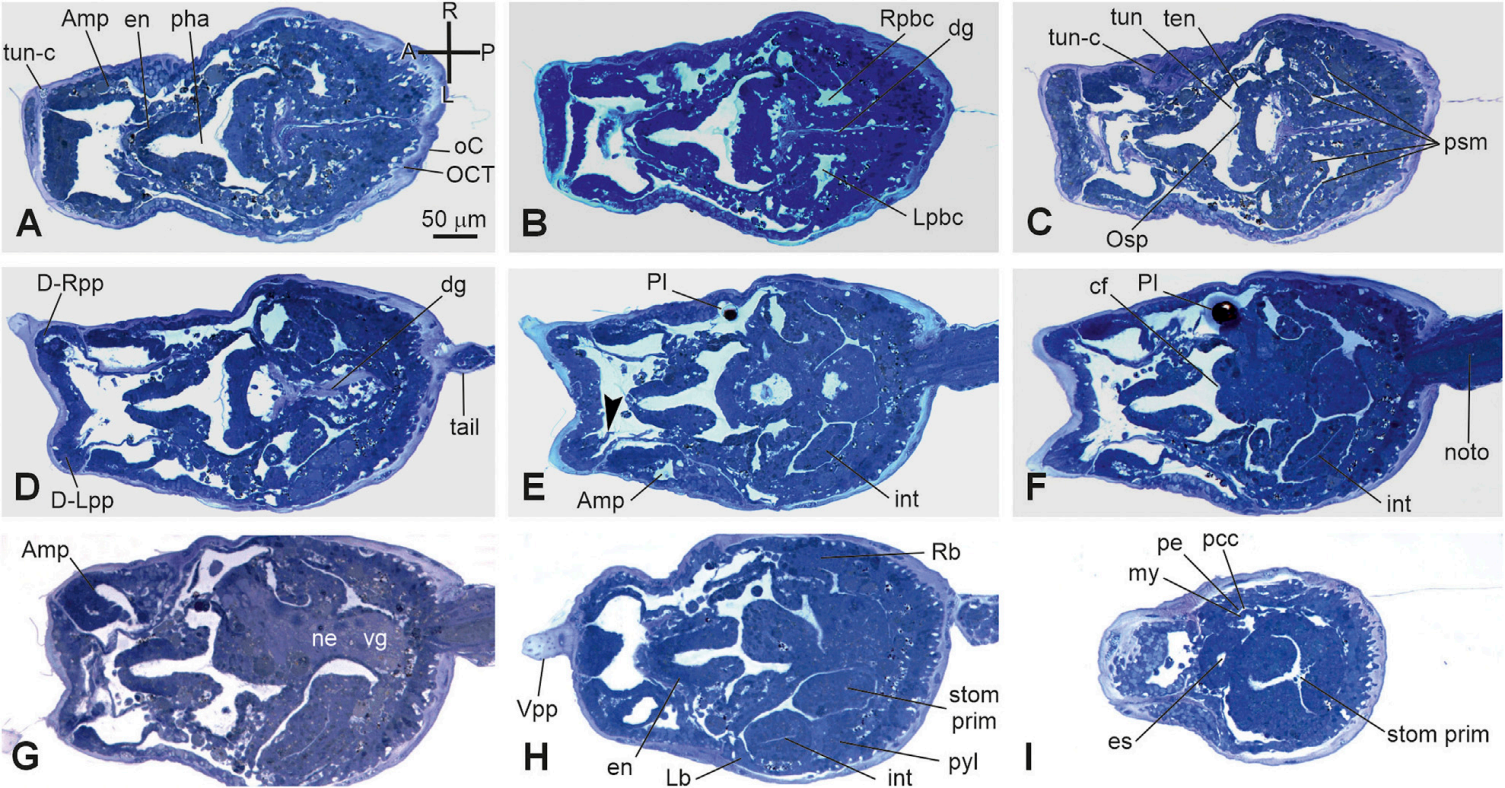
**(A-I)** VI period. Swimming larva. Stage E.6–E7.1 (Stage 18, day 6–7 of development). Frontal serial sections selected from a complete series of the same embryo cut from the dorsal side to the ventral side; E, and H are enlarged in Supplementary Figure 2G, H, respectively. Note that in H, the right bud is in the form of thickening of the peribranchial epithelium (stage 1); both the heart **(I)** and the intestine **(E–H)** are well-recognizable. The outer compartment of the tunic is populated by tunic cells **(A)**. Arrowhead in E, papillary neuron; A-P and R-L, anterior–posterior and right-left axes, respectively; Amp, ampulla; cf, ciliated funnel; dg, dorsal groove D-Lpp, dorsal left papilla; D-Rpp, dorsal right papilla; en, endostyle; int: intestine; Lb, left bud; Lpbc, left peribranchial chamber; my, myocardium; ne, neck; noto, notochord; es, esophagus; Osp, oral siphon primordium; pe, pericardium; pcc, pericardial cavity; pha, pharynx; Pl, photolith; psm, protostigma; pyl, pyloric cecum; Rb, right bud; Rpbc, right peribranchial chamber; stom prim, stomach primordium; ten, oral tentacle rudiment; tun, tunic; tun-c, tunic cell; vg, visceral ganglion; Vpp, ventral papilla. Enlargement is the same in A–I. Toluidine blue.

*Stage E6–E7.1* (Stage 18, day 6–7 of development) (Figures 2R, 3R’, Figure 7; Supplementary Figure 2G): hatched larva. The larva has a 400 mµ-long trunk and a 1 mm-long tail; it swims upward (negative to gravity) and is attracted toward light sources. Its anterior region is expanded in the eight ampullae, surrounding a central protruding area with the three papillae. Here, the receptor end-organs of the papillary neurons elongate in the anterior tunic. The eight endostyle zones differentiate (Supplementary Figure 2G; Kowarsky et al., 2021). Both the rudiments of the oral and atrial siphons face the dorsal groove; they are occluded by the tunic and will definitely open during metamorphosis. Some of the eight stomach folds are recognizable. The gut is completely enveloped by the perivisceral epithelium (Supplementary Figure 2H). The pyloric cecum is well-formed (Figure 7H). Both the left and right buds are recognizable as thickened discs of the peribranchial epithelium; the right one is wider than the left one. The pericardium and the myocardium are well-separated and delimit a narrow pericardial cavity. The larval brain is organized in a large sensory vesicle, the visceral ganglion, the neck, and the nerve cord. The adult neural complex, derived from the neurohypophyseal duct, is composed of the differentiating cerebral ganglion and neural gland complex. The yolk globules are no longer easily recognizable in the cells.

*Stage E6–E7.2* (Stage 19, day 6–7 of development) (Supplementary Figure S2): swimming larva. The larva goes through a short period of indifference to light, then, just before metamorphosis, becomes negatively photo tactic, and repeatedly touches the substrate. The receptor end-organs of the papillary neurons pass through small fenestrations in the anterior tunic protruding outside in the environment; the cells of the interpapillary region release secretions that change the properties of the tunic layers, favoring the next adhesion of the larva to the substrate.

The metamorphosing larva will definitely open the siphons after approximately 1.5 days from its adhesion (at approximately 18°C (Brunetti et al., 2017)), beginning its filtering activity. However, the oozooid morphogenesis will not be complete, and only the right bud will develop. The latter will substitute the oozooid in the filtering activity approximately 7 days from settlement and will represent the zooid (blastozooid) of the first blastogenetic generations (Brunetti et al., 2017). Gonads will mature after some blastogenetic generations (Gasparini et al., 2015), but functional germline progenitors are specified during embryogenesis (Brown et al., 2009).

### 3.3 Compared to *C. robusta*, *B. schlosseri* enhances organ development by prolonging the tailbud stages

**FIGURE 8:**
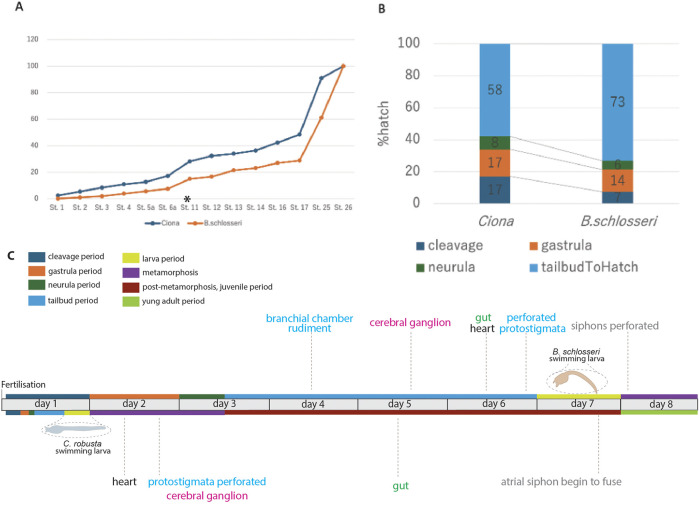
Comparison between embryonic development in *B. schlosseri* and *C. robusta*. Based on Table 2 and from the fingings of Hotta et al. (2007) and Hotta et al. (2020), **(A)** relative developmental progress of *C. robusta* and *B. schlosseri* from the cleavage stage to hatching, shown as a percentage of total time to hatching. *C. robusta* progresses through developmental stages at a much faster rate than *B. schlosseri*, particularly after the tailbud stage (start indicated with an asterisk and end corresponding to stage 26). **(B)** Percentage of total time spent at each major developmental period (cleavage, gastrula, neurula, and tailbud) in *C. robusta* and *B. schlosseri*, illustrating extended tailbud development in *B. schlosseri* compared to *C. robusta*. **(C)** Developmental timeline of *B. schlosseri* and *C. robusta*, including key anatomical milestones and periods. The colored bars represent major developmental periods/meta-periods: cleavage (blue), gastrula (orange), neurula (green), tailbud (light blue), larva (yellow), metamorphosis (purple), post-metamorphosis (brown), and young adult (olive). The timing of specific organ development such as the formation of the cerebral ganglion, heart, branchial chamber rudiment, and perforated protostigmata are indicated along the timeline.

The identification of embryonic development stages, along with the description of organ appearance and differentiation, allows for a comparison between developmental events in *B. schlosseri* and *C. robusta* (Hotta et al., 2007; Hotta et al., 2020). The two pathways differ in terms of the time taken for larval development, the relative duration of some developmental periods, the complexity of hatched larvae, and the timing of organ appearance (Figure 8).

A comparison between the percentage of time spent at each developmental stage relative to the total time from fertilization to hatching in the two species shows that the overall trend of the developmental progress from fertilization to the tailbud period is strikingly similar (Figure 8A; Table 2). However, from the tailbud period, the developmental trend between the two species diverges (Figure 8B). When examining the proportion of time spent in each developmental period (cleavage, gastrula, neurula, and tailbud periods), it was found that the tailbud period in *B. schlosseri* lasts relatively longer than the other periods.

The complexity of *B. schlosseri* larva reflects 1) the absolute extension in time of the developmental phases: at 20°C, the “Embryonic development, pre-metamorphosis” meta-period lasts 6–7 days in *B. schlosseri* and 17 h 30 min in *C. robusta* (Hotta et al., 2007; Hotta et al., 2020). Although metamorphosis and post-metamorphosis have not been yet described in detail in *B. schlosseri*, data from the literature show that these phases last 1.5 days at 18°C (Brunetti et al., 2017). In contrast, the “Metamorphosis” and “Post-metamorphosis” meta-periods in *C. robusta* last 6 days at the same temperature. 2) The relative extension in time of the tailbud period, during which the main organogenesis events occur.

Considering the larval complexity, in *B. schlosseri*, we identified 116 anatomical entities in embryos and larvae (Supplementary Table S1); only 30 of them are also exhibited by *C. robusta* during embryogenesis (Hotta et al., 2007; Hotta et al., 2020). It is notable that the anatomical ontology of *C. robusta* includes some entities not present in *B. schlosseri*, such as those related to atrium formation (atrial siphon primordia, left atrial siphon, and right atrial siphon) and sensory organs (ocellus and otolith) in the brain. These additional entities are related to anatomical differences between the two larvae and cannot be attributed to higher larval complexity in *C. robusta.* The latter also displays some larval epidermal neurons (apical trunk epidermal neurons, dorsal caudal epidermal neurons, rostral trunk epidermal neurons, and ventral caudal epidermal neurons) not described in *B. schlosseri*.

Specifically, the *B. schlosseri* embryo possesses an ectodermal dorsal groove where both the siphons open at the larval stage (although still occluded by the tunic). In particular, the atrial chamber originates from a single ectodermal mid-dorsal rudiment, as typical in Pleurogona ascidians (Manni et al., 2022; Lin et al., 2023). In contrast, *C. robusta* does not show a dorsal groove, and the oral siphon rudiment and the paired atrial siphon rudiments open independently at metamorphosis.

A photolith characterizes the larval sensory vesicle of *B. schlosseri*, as is typical for Styelidae, to which *B. schlosseri* belongs; in contrast, in most ascidian larvae, including *C. robusta*, feature a sensory vesicle containing an otolith and an ocellus. An additional vesicle, the left ganglionic vesicle, which contains a possible residuum of a primitive photoreceptor organ (Sorrentino et al., 2000), characterizes *B. schlosseri* with respect to *C. robusta*. Finally, in the tailbud period, eight ectodermal ampullae evaginate from the anterior epidermis in *B. schlosseri* for the stable adhesion of the metamorphosing larva; in *C. robusta*, a basal stalk, elongating during the “Metamorphosis” meta-period, represents the holdfast (Hotta et al., 2020).

The larval complexity, during the “Embryonic development, pre-metamorphosis” meta-period, in *B. schlosseri* is mainly due to the development of 43 prospective juvenile organs, which are absent in *C. robusta,* in addition to 73 transitory larval structures (Figure 8C; Supplementary Table S1). Organs such as the branchial chamber with perforated protostigmata, the cerebral ganglion, the heart, and the gut are recognizable in all their main subcomponents during the tailbud and larva periods in *B. schlosseri*, but these organs become recognizable only during the metamorphosis and/or the post-metamorphosis periods in *C. robusta*.

## 4 Discussion

### 4.1 Embryonic development in *B. schlosseri*

Combining complementary information from *in vivo*, CLSM, and histological analyses, our data on *B. schlosseri* embryogenesis provide insights into the species’ developmental biology, highlighting both similarities to and differences from solitary ascidians, particularly in terms of developmental timelines, features for stage identification, and anatomical structures. Moreover, these analyses offer the most accurate description of embryogenesis in a colonial ascidian so far available.

This description aligns with previous reports on *B. schlosseri* embryogenesis, which, however, only referred to a few stages or reported synthetic descriptions (Grave and Woodbridge, 1924; Grave, 1934; Scott, 1934; Manni et al., 1999; Sorrentino et al., 2000). With respect to the more recent report on *B. schlosseri* embryogenesis by Kowarsky et al. (2021), we describe a higher number of stages in this study: three gastrula period stages (E2.1, E2.2, and E2.3) instead of one (E2); two neurula period stages (E3.1 and E3.2) instead of one (E3.1); and six tailbud period stages (E3.2, E3.3, E3.4, E4, E5, and E6) instead of four (E3.2, E4, E5, and E6). The larva period, previously including one “swimming larva” stage (E6–E7), is subdivided into two stages in this study, namely, the “hatched larva” stage (E6–E7.1) and the “swimming larva” stage (E6–E7.2). Although this timeline is not precisely defined, it updates the morphogenetic atlas *Tabula compositi chordati Botrylli*, facilitating a better comprehension of the molecular signature of *B. schlosseri* embryogenesis (Kowarsky et al., 2021). Moreover, it represents a base for the future elaboration of a canonical developmental and anatomical ontology of the embryonic development in *B. schlosseri* that will complement the available ontology of blastogenetic development (Manni et al., 2014; Kowarsky et al., 2021).

Our study demonstrates that *B. schlosseri* embryos can survive and develop *ex vivo*, successfully progressing through embryonic stages to the oozooid stage when cultured outside the parental body. However, embryonic survival and development completion rates significantly varied depending on the developmental stage at the time of removal, with higher success rates observed when embryos were excised at later stages (*e.g*., tailbud period) than earlier stages (*e.g.*, gastrula period). These findings indicate that the parental environment may play a crucial role in the early stages of embryogenesis and may become less essential as development progresses, making *ex vivo* culturing more reliable in the later stages. It is, however, notable that in *B. schlosseri*, a nutrient transfer from the parent to the developing embryo through the placental cup has not been evidenced (Zaniolo et al., 1987), in contrast to closely related species such as *Botrylloides leachii* and *B. violaceus* (Zaniolo et al., 1994a; Zaniolo et al., 1998). Additionally, the differences in the developmental rate and progression of *ex vivo* embryos, particularly the accelerated development at higher temperatures (23°C), align with those of prior studies on temperature-dependent growth in *B. schlosseri* (Gasparini et al., 2015). This observation is valuable for understanding how environmental factors may impact embryogenesis and larval release timing, especially in controlled experimental settings.

Our approach to staging the *B. schlosseri* embryonic development using meta-periods, periods, and stages based on “Embryo Day” notation (*e.g.*, E.1 and E.2) provides a practical framework that compensates for the inability to accurately track the fertilization time. Although the precise timing of fertilization remains a challenge, this framework provides a reliable and consistent means of both tracking development in relation to the blastogenetic cycle and comparing embryonic progress across different species and environmental conditions. In particular, our success in culturing embryos enabled us to observe and analyze developmental processes in detail outside the parental body, overcoming the limitations posed by studying embryos solely within the protective confines of the colony. This will open new perspectives in the study of this species since some tools, such as *in vitro* embryo manipulation and transgenesis, so far not possible, could be finely tuned in the near future, allowing the advancement of comparative, developmental, and evolutionary studies. Including a high-resolution atlas of *B. schlosseri* embryonic anatomical structures, this study offers an in-depth examination of key morphological features across developmental stages, providing valuable insights into species-specific traits. Considering that in *B. schlosseri*, some developmental genes have been co-opted from embryogenesis and redeployed in the blastogenetic development (Alié et al., 2018; Kowarsky et al., 2021), this anatomical atlas will open the door to further investigations on commonalities and differences between embryogenesis, blastogenesis, and possibly regeneration.

The *B. schlosseri* larva also displays two early buds, evidencing the precocity of asexual reproduction as crucial for colony success. This highlights the presence of restricted pluripotent epithelial areas in the larva’s lateral peribranchial chamber leaflets, which are involved in budding. Stem cells in the larva may also contribute to blastogenesis as hemoblasts, *i.e.*, candidate stem cells, are detectable in the embryo, with their appearance coinciding with the high expression of hematopoietic stem cell genes (Voskoboynik et al., 2008; Rinkevich et al., 2013; Rosental et al., 2018; Kowarsky et al., 2021). Additionally, germline precursors, identified by *vasa* expression in *B. schlosseri* embryos, circulate in the colonial circulatory system for several generations before contributing to the gonad niche (Brown et al., 2009). In the larva, the right bud is more developed than the left one, evidencing the early onset of the asymmetry in the blastogenic power of the lateral body walls, as long recognized in blastozooids (Gasparini et al., 2015). Only the right bud will continue its development during the Metamorphosis and Post-Metamorphosis Periods, as typically occurs in the blastogenetic cycle when colony energy does not support bilateral growth of its buds.

### 4.2 Coloniality, heterochrony, and reproductive strategies

Allowing a direct comparison with the established developmental ontologies of *C. robusta*, this timeline bridges the gap between solitary and colonial ascidians, allowing evolutionary consideration (Hotta et al., 2007; Hotta et al., 2020).

The distinct heterochrony observed between *B. schlosseri* and *C. robusta* at both temporal (time taken to larval development and relative extension in time of some periods) and anatomical (complexity of hatched larvae and timing of organ appearance) levels underscores the evolutionary divergence within ascidians, providing clues about the adaptive significance of extended periods in *B. schlosseri*. These may be extended to other colonial ascidians, considering the unique requirements of a colonial life cycle, such as budding and colony establishment.

Although embryogenesis is not known in detail in other colonial ascidians, these species share long gestation periods coupled with high larval complexity compared to solitary ascidians (see Burighel et al. (1997) for review). This suggests that the evolution of the colonial habitus is associated with the reproductive strategy, *i.e.*, it needed a shift from oviparity to ovoviviparity/viviparity, from the external fertilization of tiny eggs to the internal fertilization of yolked eggs and from the development of orphan embryos to gestation. Coloniality in ascidians manifests in different modalities across different species and has been proposed to be the result of multiple independent acquisitions and subsequent diversifications (Alié et al., 2018). Accordingly, the variety of reproductive solutions exhibited by colonial ascidians suggests that the passage from oviparity to ovoviviparity/viviparity evolved in different ways, resulting in morpho-functional modifications of gametes (such as increased yolk amount in eggs, egg envelopes participating in the formation of placental cups/brood pouches, and specialized sperm equipped with apical structures for reaching the ovulated egg for internal fertilization), gonads (producing very few eggs per individual), oviducts (usually very short, with openings located far from the atrial aperture to facilitate the retention of embryos, and in some cases involved in the formation of the placental cups or capable of storing sperms for internal fertilization), and parent structures for housing the developing embryos (such as particular regions of the oviduct or tunic and chambers exposed to seawater) (Berrill, 1950; Zaniolo et al., 1987; Zaniolo et al., 1994a; Zaniolo et al., 1994b; Zaniolo et al., 1998; Martinucci et al., 1988; Manni et al., 1994; Burighel and Martinucci, 2000; Kawamura et al., 2011). Further comparative investigations into the morpho-functional features driving ascidian heterochrony could offer deeper insights into the evolutionary underpinnings of these developmental adaptations.

The contrasting developmental strategies of solitary and colonial tunicates, exemplified by *C. robusta* and *B. schlosseri*, offer intriguing insights into the evolutionary trade-offs between producing many larvae for open-water development and a few larvae by brooding. Although *C. robusta* prioritizes rapid development, reaching the competent larval stage within a day, its larvae hatch with less developed organs, a likely adaptation for pursuing speed and shortening the open-ocean life to avoid predation. In contrast, *B. schlosseri* larvae, protected within the parent colony, undergo slower development but hatch with more advanced organ systems, suggesting a prioritization of developmental completeness. Notably, *B. schlosseri* significantly shortens the next developmental meta-period (metamorphosis) by shifting the time normally required for the maturation of adult organs in solitary ascidians (*i.e.*, after metamorphosis) to the tailbud period. Although *C. robusta* requires only 18 h to hatch, its metamorphosis and post-metamorphosis periods extend over 6 days, allowing the organogenesis to be completed after hatching. Conversely, *B. schlosseri* spends approximately 96 h (4 days) in protected development due to ovoviviparity, hatching with mature organs and requiring only 1.5 days for metamorphosis and post-metamorphosis, a four-fold acceleration compared to *C. robusta*. These divergent strategies highlight the diverse evolutionary paths that tunicates have taken to optimize their reproductive success in different ecological contexts.

In summary, our comparative analyses of *B. schlosseri* development contribute to a deeper understanding of tunicate biology by evidencing both conserved and divergent aspects of ascidian embryogenesis. These insights also offer a framework for understanding the evolution of animal morphology and life history strategies, ultimately enhancing our comprehension of the diversity and adaptability of animal life.

## Data Availability

The datasets presented in this article are not readily available because NA. Requests to access the datasets should be directed to chiara.anselmi@unipd.it.
