# Supplementary figures and images for: Speed vs completeness: a comparative study of solitary and colonial tunicate embryogenesis
Source: Front Cell Dev Biol. 2025 Mar 11;13:1540212. doi: 10.3389/fcell.2025.1540212 (PMC11933078; doi:10.3389/fcell.2025.1540212)

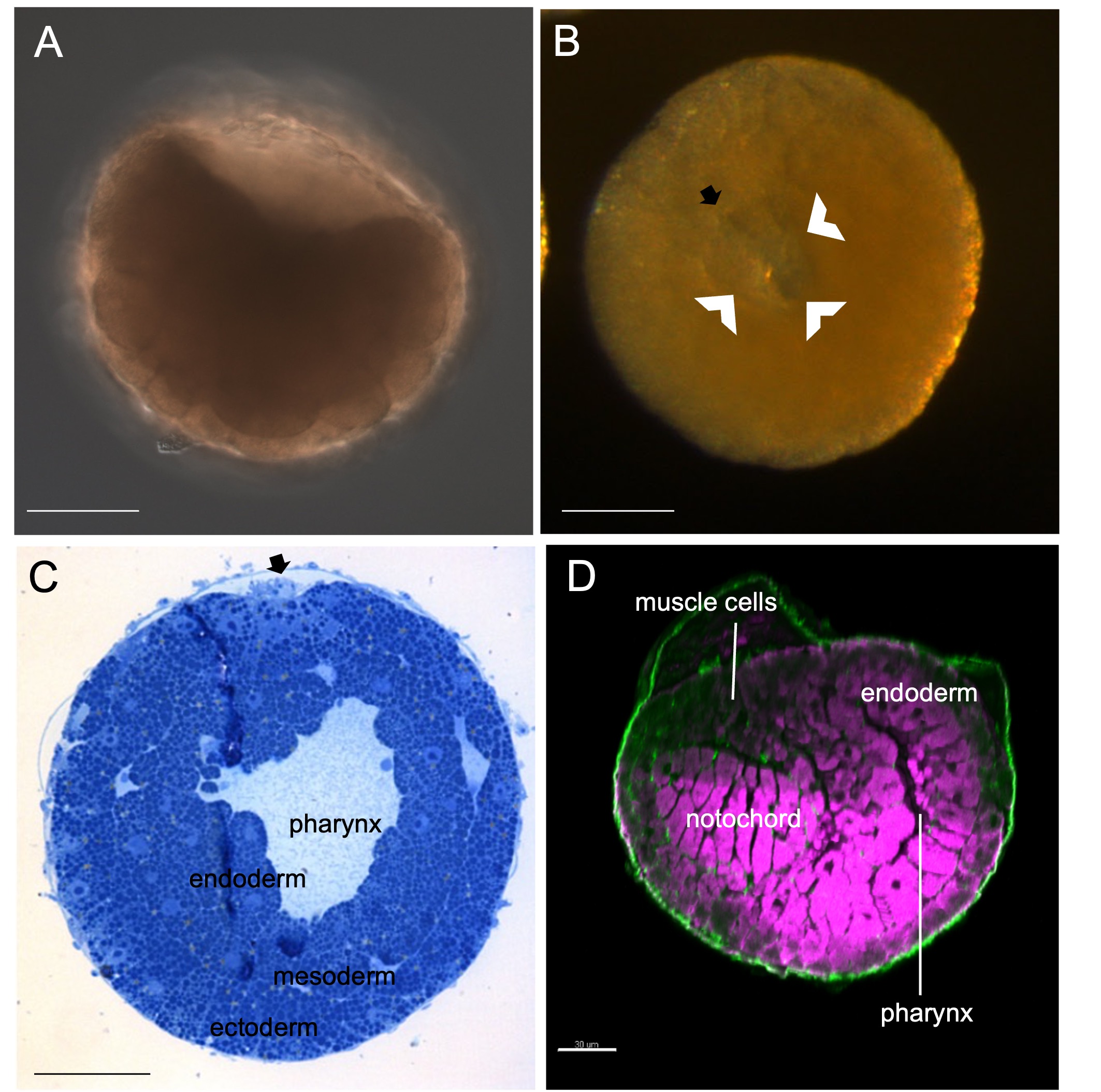

Supplement: Supplementary file 1 [file Image1.jpeg]

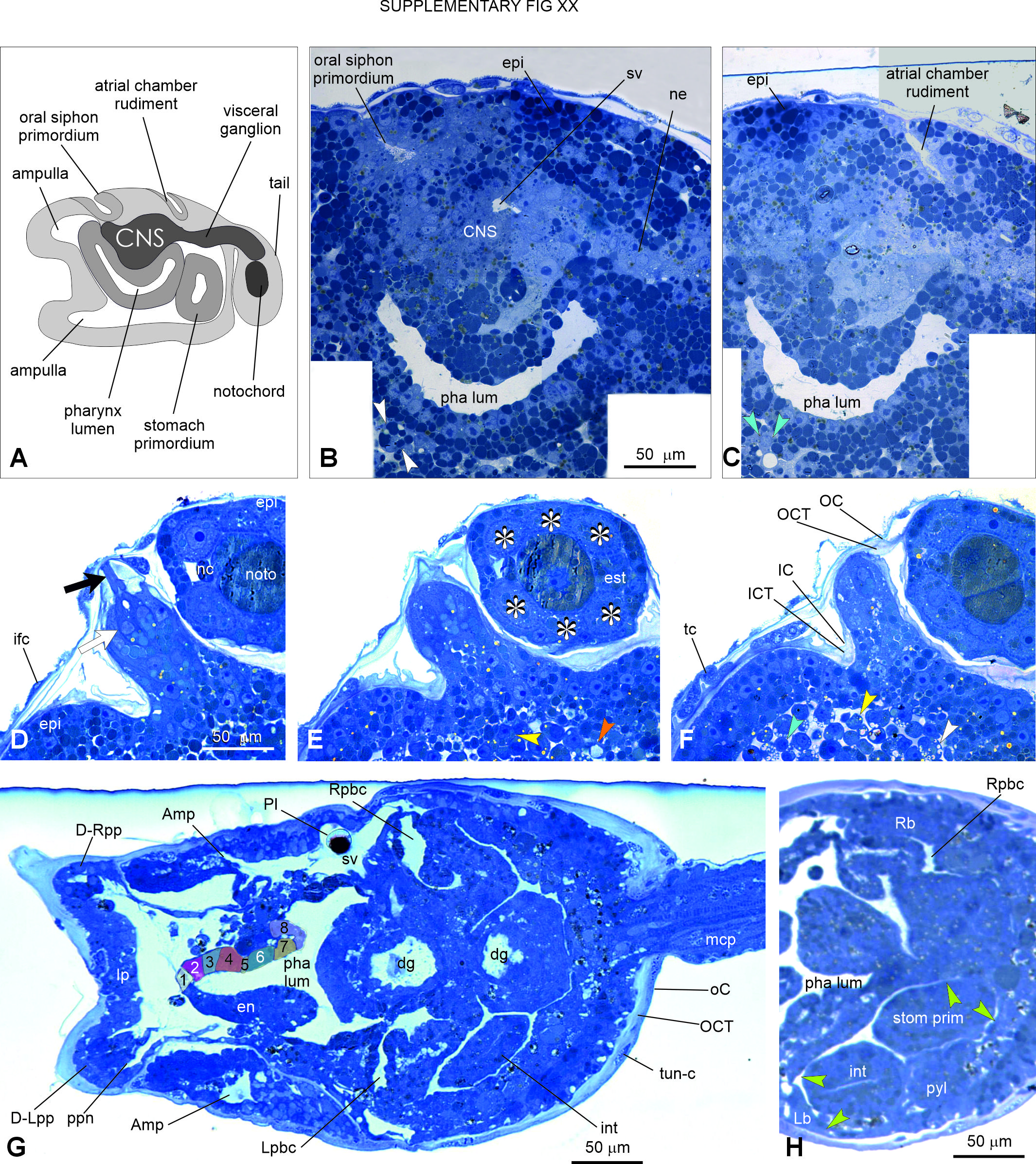

Supplement: Supplementary file 2 [file Image2.jpeg]
